# A Method for Measuring the Error Rules in Visual Inertial Odometry Based on Scene Matching Corrections

**DOI:** 10.3390/mi15111362

**Published:** 2024-11-11

**Authors:** Haiqiao Liu, Zichao Gong, Jinxu Shen, Ya Li, Qing Long

**Affiliations:** 1 The School of Electrical and Information, Hunan Institute of Engineering, Xiangtan 411104, China; liuhaiqiaoyykl@126.com (H.L.); zichao_gong@163.com (Z.G.); 70097@hnie.edu.cn (Y.L.); qinglong1018@163.com (Q.L.); 2 School of Computer Science, University of Nottingham, Nottingham NG7 2RD, UK

**Keywords:** MEMS, integrated navigation, scene matching

## Abstract

To address problems in the integrated navigation error law of unmanned aerial vehicles (UAVs), this paper proposes a method for measuring the error rule in visual inertial odometry based on scene matching corrections. The method involves several steps to build the solution. Firstly, separate models were constructed for the visual navigation model, the Micro-Electromechanical System (MEMS) navigation model, and the scene matching correction model. Secondly, an integrated navigation error measurement model based on scene matching corrections and MEMS navigation was established (the MEMS+SM model). Finally, an integrated navigation error measurement model based on scene matching corrections, visual navigation, and MEMS navigation was constructed (the VN+MEMS+SM model). In the experimental part, this paper first calculates the average error of the VN+MEMS+SM model and the MEMS+SM model under different scene matching accuracies, scene matching times, and MEMS accuracies. The results indicate that, when the scene matching accuracy is less than 10 m and the scene matching time is less than 10 s, the errors of the VN+MEMS+SM model and the MEMS+SM model are approximately equal. Furthermore, the relationship between the scene matching time and the scene matching accuracy in the EMS+SM model was calculated. The results show that, when the scene matching time is 10 s, the critical values of the image matching accuracies required to achieve average errors of 10 m, 30 m, and 50 m are approximately 160 m, 240 m, and 310 m. Additionally, when the MEMS accuracy is 150, the scene matching accuracy is 50 m, and the scene matching time exceeds 135 s, the average error of the VN+MEMS+SM model will be smaller than that of the MEMS+SM model.

## 1. Introduction

Visual navigation is a method that offers small cumulative errors and strong anti-jamming abilities and is widely used in the field of UAVs and terminal guidance [1,2]. Although MEMS navigation is cost-efficient, there is a large gap in accuracy compared with devices such as laser gyroscopes [3,4]. Therefore, the majority of researchers combine the outcomes of visual navigation and MEMS navigation through integrated navigation. However, with the increase in flight time in the process of UAV navigation, the combined visual and MEMS navigation will still lead to divergences in the navigation position results. GPS and scene matching are widely used for position correction during navigation, but it can be vulnerable to interference and deception by anti-satellite equipment, resulting in incorrect location information. On the other hand, scene matching provides a strong anti-interference ability with a low cost. Therefore, integrated navigation based on visual navigation, MEMS navigation, and scene matching correction will become the mainstream method of low-cost UAV navigation in the future [5,6].

Visual odometry can be classified into monocular and multi-eye visual odometry depending on the number of cameras used [7]. Typical monocular vision algorithms include semi-direct monocular visual odometry (SVO) [8], direct sparse odometry (DSO) [9], and ORB-SLAM based on sparse feature points [10]. DynPL-SVO [11] proposed a visual odometry suitable for dynamic scenes based on SVO and designed a dynamic mesh to identify and remove dynamic features and collect fixed point features and line features, which effectively improved the positioning accuracy. Chao et al. [12] proposed a fast direct sparse odometer scheme (DSOL) based on DSO, which processed the keyframes through the inverse compositional alignment approach and simplified the keyframes in the DSO, which effectively improved the speed of the DSO. ORB-SLAM2 [13] represented an extension of ORB-SLAM to binocular cameras, with the optimization achieved through the utilisation of the bundle adjustment (BA) method at the backend. Compared with ORB-SLAM, this method improves positioning accuracy, but it does not perform well on scenes with a weak texture, a repetitive texture, and blurry images. With advancements in deep learning, DeepVO [14] introduced a monocular end-to-end network using cyclic convolution. The DeepVO network proved that monocular VO problems can be solved in an end-to-end manner based on deep learning techniques. VIOLearner [15] proposed an unsupervised deep learning visual odometry that fused RGB-D images with inertial measurement data for absolute trajectory estimations.

In scenarios where GPS is unavailable, scene matching correction can provide the absolute position information for inertial navigation [16,17]. Therefore, the accuracy of scene matching and the time of scene matching are crucial. The traditional scene matching correction method obtained the matching pair through the ORB method [18], followed by the EPNP method [19] to calculate the position of the UAV monocular solution. Although this method is capable of achieving scene matching correction in a relatively short time, the ORB method has been observed to exhibit a suboptimal performance in more complex scenes. On this basis, the RIFT [20] method introduced phase consistency into feature point matching, but it needs more computational resources to calculate feature consistency and shows poor adaptability to changes such as rotation and scale. Based on the RIFT, Ji et al. [21] extracted the extreme points by both the maximum and minimum moments and designed the RS-GLOH descriptor, which effectively increased the number of extreme points and the number of matching pairs. However, it takes a lot of computational resources to calculate huge extreme points. R2FD2 [22] proposed a scene matching method that was robust to rotational differences. In this method, a rotation invariant feature descriptor is proposed to solve the rotation problem in remote sensing image matching, and, secondly, a rotation invariant maximum exponential map is constructed to obtain the principal direction of the extreme points. With the development of computer science, a two-stream network [23] realized scene matching by changing the image contrast and building a twin end-to-end learning network. The method took approximately 0.08 s to process a single set of remote sensing images, which offers a significant advantage over traditional methods [24]. However, its matching accuracy is lower compared to traditional methods.

Kalman filtering is the fundamental filtering method for achieving integrated navigation [25,26]. According to the fusion mode between different types of odometry, the Kalman filter can be divided into two different methods: loose coupling and tight coupling. The close-coupled system, which simultaneously inputs the data from multiple odometers into the Kalman filter for calculation, achieves a high navigation accuracy. In contrast, loosely coupled systems use inertial odometry as a predictive model and use other odometry to serve as reference observations [27]. The loosely coupled system only corrects the predicted data when the reference observations are available to ensure that the errors do not diverge [28]. Compared with tightly coupled systems, loosely coupled systems have great advantages in terms of cost and stability [29,30]. Therefore, the integrated navigation in this article adopts a loosely coupled approach for integrated navigation to explore the error propagation characteristics of the VN+MEMS+SM model and the MEMS+SM model, as well as the time of the scene matching, the accuracy of the scene matching, and the critical value of the MEMS accuracy under the determined error standard. In this paper, we will conduct an in-depth analysis of the MEMS+SM model and the VN+MEMS+SM model and calculate the critical error of integrated navigation under various working conditions. The main contributions of this paper are as follows:

(1) An integrated navigation error measurement model depending on scene matching correction and MEMS navigation (MEMS+SM model) is proposed. An integrated navigation error measurement model based on scene matching correction, visual navigation, and MEMS navigation is constructed (VN+MEMS+SM model).

(2) The critical value of the scene matching accuracy required to achieve different average errors in the MEMS+SM model and the critical value of the average error in the VN+MEMS+SM model is lower than that of the MEMS+SM model.

This paper is divided into four parts. The first part is the introduction, which introduces the current status of visual navigation, MEMS navigation, and scene matching correction methods. The second part introduces how to build the MEMS+SM model and the VN+MEMS+SM model. The third part is the experiment and discussion part; finally, there are the conclusions.

## 2. VN+MEMS+SM Model and MEMS+SM Model

**Figure 1:**
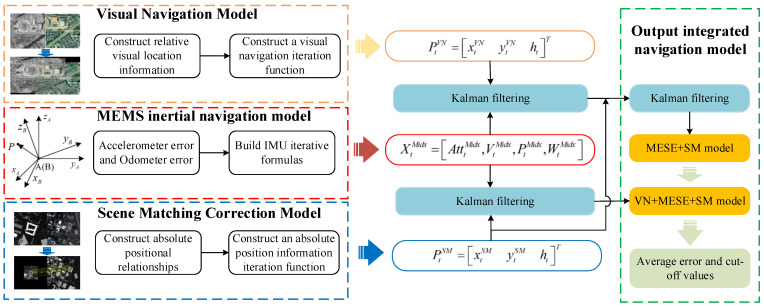
Total flow chart.

The overall flow chart of this article is shown in Figure 1. In this paper, three basic navigation models will be introduced: the visual navigation model, the MEMS inertial navigation model, and the scene matching correction model. According to the combination of these three basic models and the Kalman filter, two integrated navigation models are then constructed. Finally, the average error and the critical value of the navigation error are analysed through these two integrated navigation models.

In Figure 1, $X_{t}^{Midx}=\left[Att_{t}^{Midx},V_{t}^{Midx},P_{t}^{Midx},W_{t}^{Midx}\right]$ is the inertial navigation state quantity calculated by the MEMS IMU model, $Att_{t}^{Midx}$ represents the triaxial attitude angle, $V_{t}^{Midx}$ represents the triaxial velocity value, $P_{t}^{Midx}$ represents the triaxial position coordinates, and $W_{t}^{Midx}$ represents the triaxial angular velocity. $P_{t}^{VN}={\left[\begin{array}{ccc} x_{t}^{VN} & y_{t}^{VN} & h_{t} \end{array}\right]}^{T}$ represents the three-axis relative position coordinates calculated by the visual navigation model. $P_{t}^{SM}={\left[\begin{array}{ccc} x_{t}^{SM} & y_{t}^{SM} & h_{t} \end{array}\right]}^{T}$ represents the three-axis absolute position coordinate calculated by the scene matching correction model. For ease of understanding, Table 1 lists some of the key variables in this article and their descriptions.

### 2.1. Construction of the Basic Model and Trajectory Truth

#### 2.1.1. Trajectory Truth

In this paper, the true value of the trajectory is constructed by setting the angular velocity, acceleration, and duration of the three axes. Suppose the state quantity is $\left[Att,V,P,W,A\right]$, where Att represents the triaxial attitude angle vector; V represents the triaxial velocity vector; P represents a triaxial position vector; W represents the triaxial angular velocity vector; and A stands for a three-axis acceleration vector. If the initial state of the aircraft is expressed as $\left[Att_{0},V_{0},P_{0},W_{0},A_{0}\right]$, for the next stage, the recursive formula for the state of truth is as follows.

(1) $$\left[\begin{array}{c} Att_{t+\Delta t}\\ V_{t+\Delta t} \end{array}\right]=\left[\begin{array}{c} Att_{t}\\ V_{t} \end{array}\right]+\left[\begin{array}{c} W_{t}\cdot \Delta t\\ f_{c}\left(A_{t}\right)\cdot \Delta t \end{array}\right]$$

(2) $$P_{t+\Delta t}=P_{t}+\frac{V_{t}+V_{t+\Delta t}}{2}\cdot \Delta t$$

In the formula, $W_{t}$ is the triaxial angular velocity in the current period, $f_{c}\left(A_{t}\right)$ is the conversion equation from the coordinate system of the aircraft body to the earth coordinate system, and $A_{t}$ represents the triaxial acceleration vector in the current period. $Att_{t+\Delta t}$, $V_{t+\Delta t}$, and $P_{t+\Delta t}$ represent the updated three-axis state quantity. $Att_{t}$, $V_{t}$, and $P_{t}$ represent the current state quantity, and Δt is the solution step. At this point, by setting $W_{t}$ and $A_{t}$ for each time sample, the true state amount of each moment $X_{t}=\left[Att_{t},V_{t},P_{t},W_{t}\right]$ during the flight can be obtained. The corner t represents a different solution time.

#### 2.1.2. MEMS Inertial Navigation Model

The accuracy of the MEMS is influenced by the gyroscope constant drift, gyroscope angular walk coefficient, accelerometer constant drift, and accelerometer walk coefficient. For the convenience of the model construction and to obtain the MEMS IMU accuracy under different error states, this model constructs the MEMS IMU model under a variety of operating conditions by setting a threshold and multiplying it by the corresponding coefficient. In the MEMS inertial navigation model, a different accuracy is simulated by adding errors to the diagonal velocity and acceleration. The error formula for the angular velocity $W_{t}^{Midx}$ and the acceleration $V_{t}^{Midx}$ is shown below.

(3) $$W_{t}^{Midx}=W_{t}+Ac^{Midx}\cdot \left(eb\cdot \Delta t+\delta W_{eb}\right)$$

(4) $$V_{t}^{Midx}=V_{t}+Ac^{Midx}\cdot \left(db\cdot \Delta t+\delta V_{db}\right)$$

where $Ac^{Midx}$ is the set of the input threshold points and Midx is the index of the threshold point set. eb is the gyroscope bias transfer parameter: eb=0.01 deg/h. db is the accelerometer bias transfer parameter: db=100 μg/Hz. δ is a random number between 0 and 1 and is used to simulate the random walk coefficient. $W_{eb}$ is the maximum wander error of the gyroscope: $W_{eb}=0.001$ deg/h. $V_{db}$ is the maximum walk error of the accelerometer: $V_{db}=10$ μg/Hz. If the initial state quantity is expressed as $\left[Att_{0},V_{0},P_{0},W_{0},A_{0}\right]$ in the MEMS inertial navigation model, for the next stage, the recursive formula for the state of truth is as follows.

(5) $$\left[\begin{array}{c} Att_{t+\Delta t}^{Midx}\\ V_{t+\Delta t}^{Midx} \end{array}\right]=\left[\begin{array}{c} Att_{t}^{Midx}\\ V_{t}^{Midx} \end{array}\right]+\left[\begin{array}{c} W_{t}^{Midx}\cdot \Delta t\\ f_{c}\left(A_{t}\right)\cdot \Delta t \end{array}\right]$$

(6) $$P_{t+\Delta t}^{Midx}=P_{t}+\frac{V_{t}^{Midx}+V_{t+\Delta t}^{Midx}}{2}\cdot \Delta t$$

From the above equation, the three-axis state quantity $X_{t}^{Midx}=\left[Att_{t}^{Midx},V_{t}^{Midx},P_{t}^{Midx},W_{t}^{Midx}\right]$ calculated by the MEMS inertial navigation model can be obtained.

#### 2.1.3. Visual Navigation Model

In visual navigation, a visual odometer is constructed by matching a sequence of images. For UAVs, the model realizes visual navigation through a sequence image that is taken downward. However, it is challenging for the UAV to maintain an absolute orthogonal downward view during the flight, leading to certain errors in the matching algorithm [23,31]. As a result, the visual odometry errors accumulate with the image matching error between each frame. In this model, the cumulative error is added to the position truth to simulate the error that exists during the actual flight. The formula for the visual odometer is as follows.

(7) $$\left[\begin{array}{c} x_{t+\Delta tVN}^{VN}\\ y_{t+\Delta tVN}^{VN} \end{array}\right]=\left[\begin{array}{c} x_{t}^{VN}\\ y_{t}^{VN} \end{array}\right]+\left[\begin{array}{c} dpos\\ dpos \end{array}\right]$$

In Equation (7), $x_{t+\Delta tVN}^{VN}$ and $y_{t+\Delta tVN}^{VN}$ represent the positions of the up and down states in the *X*-axis and *Y*-axis directions, respectively, and $x_{t}^{VN}$ and $y_{t}^{VN}$ represent the positions in the *X*-axis and *Y*-axis directions, respectively. ΔtVN is the solution period of the visual navigation. dpos is the error. Due to the large error in the height information calculated by the UAV vision, the absolute altitude information $h_{t}$ is obtained by using the barometric altimeter in this model. From this, we can derive the position state quantity $P_{t}^{VN}={\left[\begin{array}{ccc} x_{t}^{VN} & y_{t}^{VN} & h_{t} \end{array}\right]}^{T}$ of the visual navigation.

#### 2.1.4. Scene Matching Correction Model

The methodology of scene matching is employed to eliminate the cumulative errors that occur during navigation. First, scene matching is performed by aligning real-time drone images with a reference map. Then, the EPNP method is applied to solve for the pose and obtain the absolute position information of the drone.

Scene matching comprises three fundamental networks [21]: the feature extraction network, the feature fusion network, and the affine matrix generation network. In the feature extraction network, the SE-ResNeXt101 is employed to extract the depth information from both the reference and real-time images, obtaining feature matrices. In the feature fusion network, matrix multiplication is applied to acquire the fused features of the image set. In the affine matrix generation network, convolutional and fully connected layers are used to obtain the affine matrix. The network uses the root mean square error (RMSE) as the loss function, calculated as shown below.

(8) $$l=\frac{1}{N}{\sum_{i,j=1}^{N}d\left(M_{1}\left(x_{i},y_{j}\right),M_{2}\left(x_{i},y_{j}\right)\right)}^{2}$$

In the above formula, $\left(x_{i},y_{j}\right)$ represents a set of points selected from the image. In the collected image, one point is selected every 20 pixels, and the total number of selected points is N. $M_{1}\left(x_{i},y_{j}\right)$ represents the result obtained using the predicted affine matrix, $M_{2}\left(x_{i},y_{j}\right)$ represents the result obtained using the true affine values, and $d\left(M_{1}\left(x_{i},y_{j}\right),M_{2}\left(x_{i},y_{j}\right)\right)$ is the Euclidean distance matrix calculated between the corresponding point sets of the two results.

Due to the potential prediction errors in the network, one of the key aspects of scene matching is the accuracy of registration. To simulate matching accuracy under various conditions, the scene matching correction model incorporates the errors that exist during actual flight by adding errors to the ground truth of the position. The calculation formula is as follows.

(9) $$\left[\begin{array}{c} x_{t}^{SM}\\ y_{t}^{SM}\\ h_{t} \end{array}\right]=\left[\begin{array}{c} x_{t}\\ y_{t}\\ h_{t} \end{array}\right]+\left[\begin{array}{c} x_{idx}+x_{idx}\times rand\left(1\right)\\ y_{idx}+y_{idx}\times rand\left(1\right)\\ 0 \end{array}\right]$$

In the above equation, $\left[\begin{array}{ccc} x_{t} & y_{t} & h_{t} \end{array}\right]=P_{t}$. $P_{t}$ is the truth value of the position at the current time. $x_{t}^{SM}$ and $y_{t}^{SM}$ represent the position information of the drone in the X and Y directions, respectively. $x_{idx}$ and $y_{idx}$ are artificially given error values, respectively. $rand\left(1\right)$ is used to randomly generate a random function between 0 and 1. From this, it can be determined that the scene matches the absolute position state quantity $P_{t}^{SM}={\left[\begin{array}{ccc} x_{t}^{SM} & y_{t}^{SM} & h_{t} \end{array}\right]}^{T}$.

Another key aspect of scene matching is the matching time. The processing time for a single image using deep learning-based scene matching is approximately 8 ms [23]. To simulate varying scene matching times under different working conditions, this study introduces a reserved scene matching time parameter, Time, measured in seconds.

### 2.2. MEMS+SM Model

In this method, the integrated navigation of the MEMS inertial navigation model and scene matching correction model is achieved by Kalman filter loose coupling. The time interval of the scene matching correction model is denoted as $\Delta tSM_{idx}$; the MEMS+SM model simulates the scene matching correction at different time intervals; and idx is the array index. At the moment when there is a scene matching correction, the formula for calculating the estimated value $\Delta X_{t+\Delta t}^{MI+SM}$ of the integrated navigation error for the next period is as follows.

(10) $$\Delta P_{t}^{SM}=P_{t}^{SM}-P_{t}^{Midx}$$

(11) $$\left(\begin{array}{cc} \Delta X_{t+\Delta t}^{MI+SM} & Kk_{t} \end{array}\right)=f_{kalman}\left(\begin{array}{cc} X_{t}^{Midx} & \begin{array}{cc} \Delta P_{t}^{SM} & H_{k} \end{array} \end{array}\right)$$

In the above equation, $H_{k}$ is the observation matrix and $Kk_{t}$ is the Kalman gain in the current state. At the moment when there is no scene matching correction, the integrated navigation error state estimator $\Delta X_{t+\Delta t}^{MI+SM}$ and the predicted state covariance matrix $U_{t+\Delta t}$ for the next period are calculated. The calculation formula is as follows.

(12) $$\left(\begin{array}{cc} \Delta X_{t+\Delta t}^{MI+SM} & Kk_{t} \end{array}\right)=f_{kalman}\left(X_{t}^{Midx}\right)$$

The formula for calculating the iterative parameters of the Kalman filter is shown below.

(13) $$Kk_{t+\Delta t}=U_{t}{\left(H_{k}\right)}^{T}\cdot {\left(H_{k}U_{t}{\left(H_{k}\right)}^{T}+R_{k}\right)}^{-1}$$

(14) $$U_{t+\Delta t}=\left(1-Kk_{t+\Delta t}H_{k}\right)U_{t}$$

In the above equation, $R_{k}$ is the covariance matrix of the measured noise, $U_{t}$ is the covariance matrix in the current state, $U_{t+\Delta t}$ is the covariance matrix of the predicted state at the next moment, and $Kk_{t+\Delta t}$ is the Kalman gain.

By comparing the difference between the MEMS inertial navigation solution value $X_{t}^{Midx}$ and the true value $X_{t}$, the error truth value $\Delta X_{t}^{Midx}$ of the MEMS inertial navigation model is obtained. After being compared with the integrated navigation error estimate $\Delta X_{t}^{MI+SM}$, the error value $err^{MI+SM}$ of the MEMS+SM model can be obtained. The calculation formula is as follows.

(15) $$\Delta X_{t}^{Midx}=X_{t}^{Midx}-X_{t}$$

(16) $$err^{MI+SM}=abs\left(\Delta X_{t}^{MI+SM}-\Delta X_{t}^{Midx}\right)$$

### 2.3. VN+MEMS+SM Model

The VN+MEMS+SM model integrates the combination of an MEMS inertial navigation model, a visual navigation model, and a scene matching correction model through two Kalman filters. The first Kalman filter combines an MEMS inertial navigation model and a vision model, while the second Kalman filter incorporates the results of the scene matching correction model into the combination of the previous step.

In the first Kalman filter, at the moment when there is a visual navigation odometer, the formula for calculating the integrated navigation error estimate $\Delta X_{t+\Delta t}^{MI+VN}$ for the next period is shown below.

(17) $$\Delta P_{t}^{VN}=P_{t}^{VN}-P_{t}^{Midx}$$

(18) $$\left(\begin{array}{cc} \Delta X_{t+\Delta t}^{MI+VN} & Kk_{t} \end{array}\right)=f_{kalman}\left(\begin{array}{cc} X_{t}^{Midx} & \begin{array}{cc} \Delta P_{t}^{VN} & H_{k} \end{array} \end{array}\right)$$

At the moment when there is no visual odometer, the formula for calculating the estimated value $\Delta X_{t+\Delta t}^{MI+VN}$ of the integrated navigation error for the next cycle is as follows. For the iterative parameters of the Kalman filter, refer to Equations (11) and (12).

(19) $$\left(\begin{array}{cc} \Delta X_{t+\Delta t}^{MI+VN} & Kk_{t} \end{array}\right)=f_{kalman}\left(X_{t}^{Midx}\right)$$

In the second Kalman filter, the model simulates the scene matching correction at different time intervals, and the time interval of the scene matching correction is $\Delta tSM_{idx}$. At the moment when there is a scene matching correction, the formula for calculating the estimated value $\Delta X_{t+\Delta t}^{MI+VN+SM}$ of the integrated navigation error for the next period is as follows.

(20) $$\Delta P_{t}^{SM-VN}=P_{t}^{SM}-P_{t}^{VN}$$

(21) $$\left(\begin{array}{cc} \Delta X_{t+\Delta t}^{MI+VN+SM} & Kk_{t} \end{array}\right)=f_{kalman}\left(\begin{array}{cc} X_{t}^{Midx} & \begin{array}{cc} \Delta P_{t}^{SM-VN} & H_{k} \end{array} \end{array}\right)$$

At the moment when there is no scene matching correction, the formula for calculating the estimated value $\Delta X_{t+\Delta t}^{MI+VN+SM}$ of the integrated navigation error for the next period is as follows. For the iterative parameters of the Kalman filter, refer to Equations (11) and (12).

(22) $$\left(\begin{array}{cc} \Delta X_{t+\Delta t}^{MI+VN+SM} & Kk_{t} \end{array}\right)=f_{kalman}\left(X_{t}^{Midx}\right)$$

By comparing the error estimation value $\Delta X_{t}^{MI+VN}$ of the MEMS inertial navigation and visual integrated navigation with the error truth value $\Delta X_{t}^{Midx}$ of the MEMS inertial navigation model, the error truth value $err^{MI+VN}$ of the MEMS and visual integrated navigation can be obtained. The calculation formula is as follows.

(23) $$err^{MI+VN}=abs\left(\Delta X_{t}^{MI+VN}-\Delta X_{t}^{Midx}\right)$$

The true error $err^{MI+VN+SM}$ of the VN+MEMS+SM model can be compared with the error estimation $\Delta X_{t}^{MI+VN}$ of the MEMS and visual integrated navigation model and the true error of the MEMS inertial navigation model: $\Delta X_{t}^{Midx}$ and $\Delta X_{t+\Delta t}^{MI+VN+SM}$. The calculation formula is as follows.

(24) $$err^{MI+VN+SM}=abs\left(\Delta X_{t+\Delta t}^{MI+VN+SM}-abs\left(\Delta X_{t}^{MI+VN}-\Delta X_{t}^{Midx}\right)\right)$$

## 3. Simulated Experiment and Discussion

The integrated navigation code uses Matlab 2018a (9.4.0.813654) as a software simulation platform. A set of flight trajectory truth values $X_{t}=\left[Att_{t},V_{t},P_{t},W_{t}\right]$ were generated by Matlab. The total flight time was 1000 s. And the solution step of the MEMS inertial navigation model was 0.1 s. In this part, the VN+MEMS+SM model and the MEMS+SM model will be analysed, and the cut-off values for the corresponding models will be calculated separately. The triaxial angular velocity and the amount of triaxial acceleration are recorded in Table 2 during the flight of the UAV model.

In Table 2, Phase 1 is the acceleration phase, Phase 2 and Phase 6 are left turns, Phase 3 and Phase 5 are right turns, and Phase 4 and Phase 7 maintain a constant speed.

**Figure 2:**
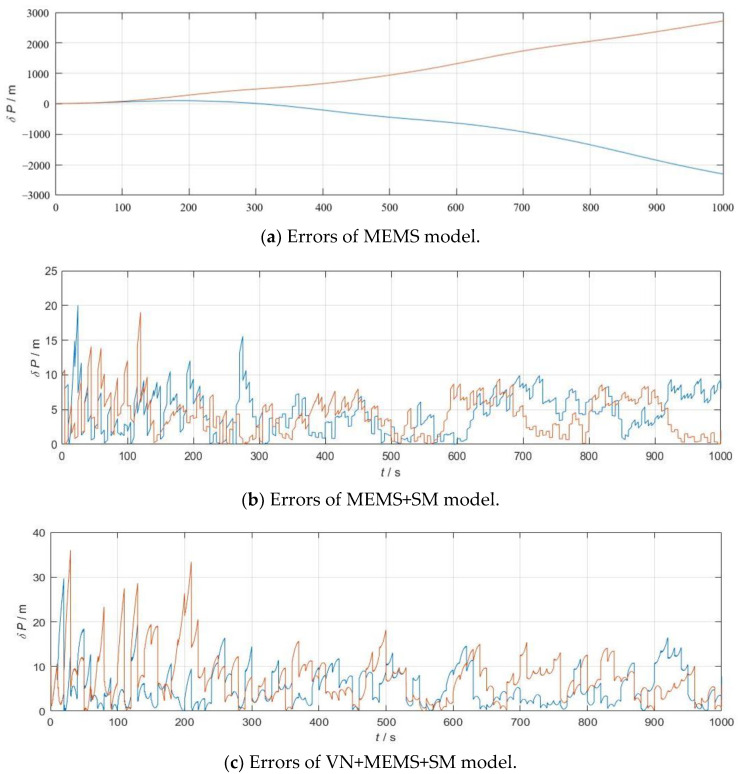
Errors of different models.

To demonstrate that integrated navigation can effectively suppress the generation of MEMS errors, Figure 2 shows the error results for the X-axis and Y-axis under the same conditions for the MEMS model, MEMS+SM model, and VN+MEMS+SM model.

In Figure 2, the MEMS accuracy used is 10, with the horizontal axis representing flight time and the vertical axis indicating the cumulative errors during the flight. The blue lines in the image represent deviations in the X-axis direction, while the yellow lines represent deviations in the Y-axis direction. Figure 2a demonstrates that, with this level of accuracy, the error continues to increase over time. In Figure 2b, the scene matching reservation time is 5 s, with the scene matching accuracy being 10 m. In Figure 2c, the visual navigation solution cycle is 1 s, with a scene matching reservation time of 5 s and a scene matching accuracy of 10 m. It can be observed that both the VN+MEMS+SM model and the MEMS+SM model illustrate significantly reduced cumulative errors. The subsequent section will further explore the average errors of the VN+MEMS+SM model and the MEMS+SM model under various working environments.

### 3.1. Error Analysis of the MEMS+SM Model

**Figure 3:**
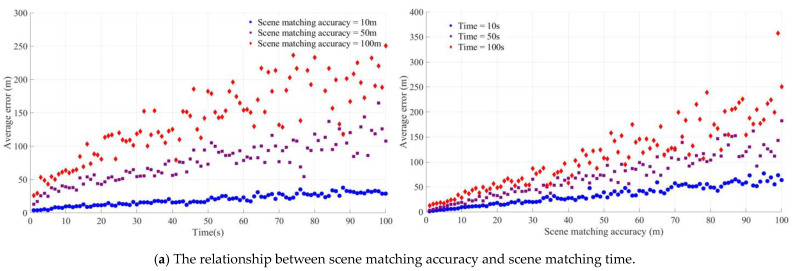

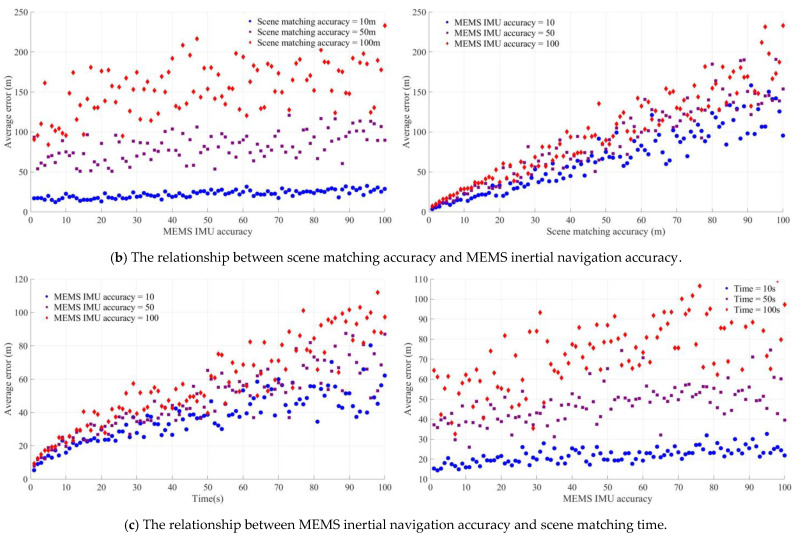
The average error of the MEMS+SM model under different working conditions.

The accuracy of the MEMS inertial navigation, image matching accuracy, and image matching time are the critical factors affecting the final integrated navigation accuracy. In Figure 3, the matching time refers to the reserved computation time for matching. We simulated matching algorithms with varying computation times by reserving different amounts of time. Figure 3 illustrates the average error values of the model under different operating conditions.

Figure 3a illustrates the trend of the average error with the scene matching correction time and the average error with the scene matching accuracy when the MEMS inertial navigation accuracy is 50. When the scene matching accuracy is 100 m, the average error is approximately 25 m, and, when the scene matching accuracy is 100 m, the average error is 250 m. Compared with the same case with an elephant matching accuracy of 10 m and 50 m, the divergence is the most significant.

Figure 3b illustrates the trend of the average error with the MEMS inertial navigation accuracy and the average error with the scene matching time when the scene matching accuracy is 30 m. When the scene matching accuracy is 10 m, the average error of the MEMS inertial navigation remains between 10 m and 30 m, regardless of the changes in the accuracy of the MEMS inertial navigation changes. Although the average error of the scene matching accuracy is between 50 m and 100 m, the average error also remains within a certain range.

Figure 3c demonstrates the trend of the average error with the MEMS inertial navigation accuracy and the average error with the scene matching accuracy when the scene matching time is 60 s. It can be observed that, when the scene matching accuracy is determined, changing the scene matching time will influence the average accuracy, but, when the matching time is less than 10 s, the average error is independent of and less than the MEMS inertial navigation accuracy. The accuracy of the visible scene matching and the time of the scene matching are essential for the error correction of the MEMS inertial navigation. The optimal balance between the accuracy of the scene matching and the timing of the scene matching will be explored below.

### 3.2. Threshold of the MEMS+SM Model

**Figure 4:**
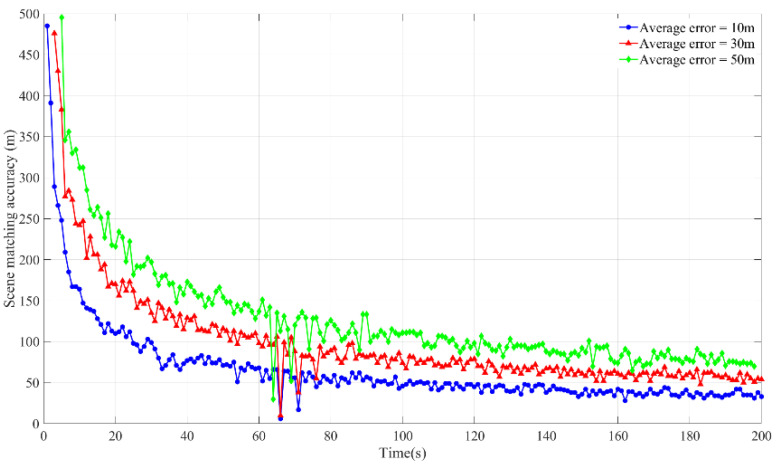
The critical value of the scene matching error at different scene matching times.

To further explore the critical value of the MEMS+SM model, the critical value of the scene matching accuracy is calculated when varying degrees of average error are reached under different matching times, and the results are shown in Figure 4.

In Figure 4, the critical values of scene matching accuracy and time, with the horizontal axis representing the time of image matching and the vertical axis being the accuracy of scene matching, are shown when the average error is 10 m, 30 m, and 50 m and the MEMS inertial navigation accuracy is 50. When the image matching time is 10 s, the critical values of the image matching accuracy required to achieve the average errors of 10 m, 30 m, and 50 m are about 160 m, 240 m, and 310 m. When the time of the scene matching is less than 10 s, the average error exhibits minimal change as the accuracy value of the scene matching increases. Similarly, when the error of the scene matching is less than 20 m, the average error changes less as the time required for the scene matching increases.

### 3.3. Error Analysis of the VN+MEMS+SM Model

**Figure 5:**
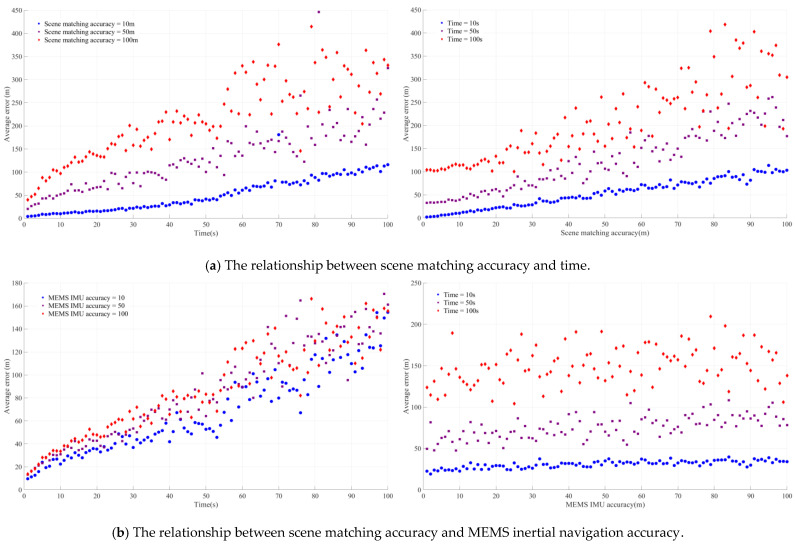

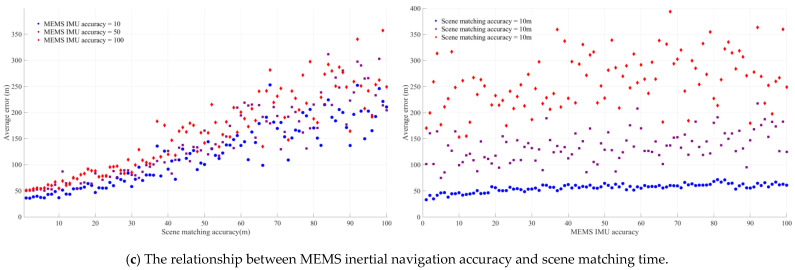
The average error of the VN+MEMS+SM model under different working conditions.

This method also explores the influence of the MEMS inertial navigation accuracy, image matching accuracy, and image matching time on the model. Figure 5 shows the mean error values of the model under diverse operational conditions.

Figure 5a represents the trend of the average error with the scene matching correction time and the average error with the scene matching accuracy when the MEMS inertial navigation accuracy is 50. When the scene matching accuracy is 10 m, the scene matching time changes from 1 s to 100 s, and the average error increases from 1 m to approximately 100 m. When compared to the results in Figure 3a, the average error increases from 1 m to about 40 m in the same situation. It is evident that the average error of the VN+MEMS+SM model in the high-precision region is much higher than that of the MEMS+SM model.

Figure 5b displays the trend of the average error with the accuracy of the MEMS inertial navigation and the trend of the average error with the scene matching time when the scene matching accuracy is 30 m. A similar conclusion can be drawn from Figure 3b, where the average error remains between 10 m and 30 m, regardless of the change in the accuracy of the MEMS inertial navigation, and the scene matching accuracy is 10 m. Comparing the case in Figure 3a, it can be concluded that, when the scene matching accuracy is high, the average errors generated by the VN+MEMS+SM model and the MEMS+SM model are about the same.

Figure 5c shows the trend of the average error with the MEMS inertial navigation accuracy and the average error with the scene matching accuracy when the scene matching time is 60 s. The results show that, when the matching time is less than 10 s, the average error of the MEMS inertial navigation is less than 25 m, regardless of the change in the accuracy of the MEMS inertial navigation. A comparison of these errors with those in Figure 3c indicates that the average errors produced by the VN+MEMS+SM model and the MEMS+SM model are similar when the scene matching time is small. Therefore, this paper explores the error law of the VN+MEMS+SM model and the MEMS+SM model under a large image matching time.

### 3.4. Threshold of the VN+MEMS+SM Model

**Figure 6:**
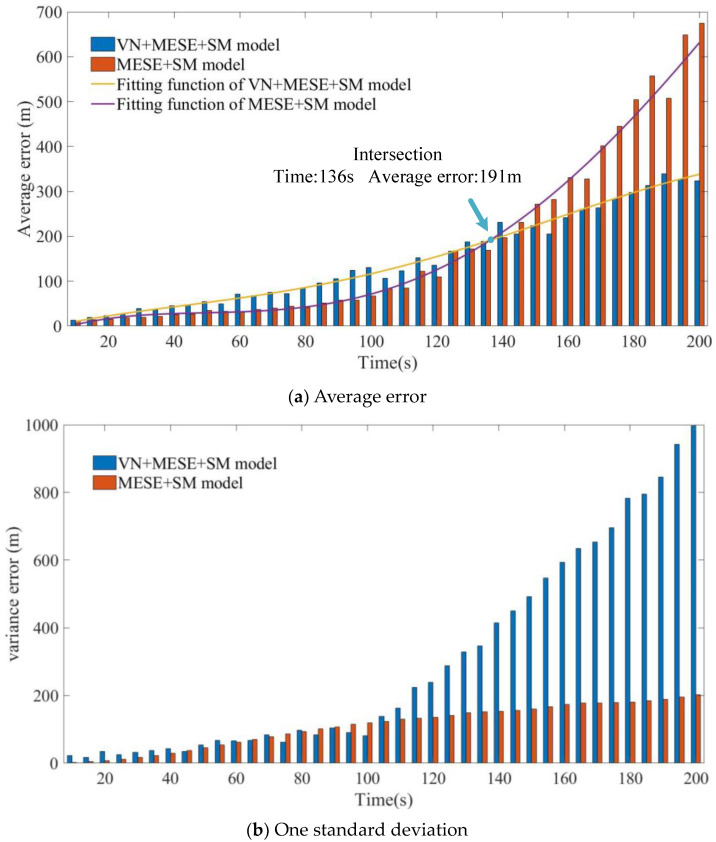
The average error and one standard deviation of the VN+MEMS+SM model and MEMS+SM model.

Figure 6 shows the variation of the average error of the VN+MEMS+SM model and the MEMS+SM model with the scene matching error time when the MEMS inertial navigation accuracy is 150, while the scene matching accuracy is 50 m.

In Figure 6a, when the MEMS inertial navigation accuracy and the scene matching accuracy are equivalent and the scene matching time is less than 135 s, the error of the VN+MEMS+SM model is greater than that of the MEMS+SM model when the matching time is about 135 s. The error of the VN+MEMS+SM model is equal to the error of the MEMS+SM model when the matching time is greater than 135 s. It can be seen that, with the MEMS inertial navigation accuracy being equal to 150, the scene matching accuracy is reduced to 50 m. While the scene matching time is 135 s, the VN+MEMS+SM model is equal to the MEMS+SM model, and, when the scene matching time is greater than 135 s, the average error of the VN+MEMS+SM model will be less than that of the MEMS+SM model. To better fit the discrete points, the method uses the fourth polynomial to fit the error $err^{MI+VN+SM}$ of the VN+MEMS+SM model and the error $err^{MI+SM}$ of the MEMS+SM model.

As illustrated in Figure 6b, the incorporation of visual odometry results in a reduction in the variance of the VN+MEMS+SM model in comparison to the MEMS+SM model. Although visual odometry also introduces errors, it is still capable of correcting errors compared to the MEMS at this level of accuracy. In the MEMS+SM model, as the scene matching correction time increases, the MEMS errors increase exponentially, causing the standard deviation to increase correspondingly in an exponential manner. In the case of long correction times and a low MEMS accuracy, the errors will diverge rapidly. Although the average error is reduced by the scene matching correction, the dispersion of the data remains considerable, leading to a labelling difference that is greater than the average error.

To explore the critical value of the scene matching time when the errors of the VN+MEMS+SM model and MEMS+SM model are equal under different MEMS inertial navigation accuracies, the average error of the two models under different working conditions is calculated first, and the four-order function is used for the fitting. Second, the intersection points of the fitting function are, then, calculated. This is the critical time corresponding to the current MEMS inertial navigation accuracy. Figure 6 shows the critical relationship between the MEMS inertial navigation accuracy and time in the case of 10 m, 30 m, and 50 m scene matching correction accuracies, and the average error of the MEMS+SM model in the area above the curve is greater than that of the VN+MEMS+SM model. The area below the curve represents the average error of the MEMS+SM model, which is less than that of the VN+MEMS+SM model.

**Figure 7:**
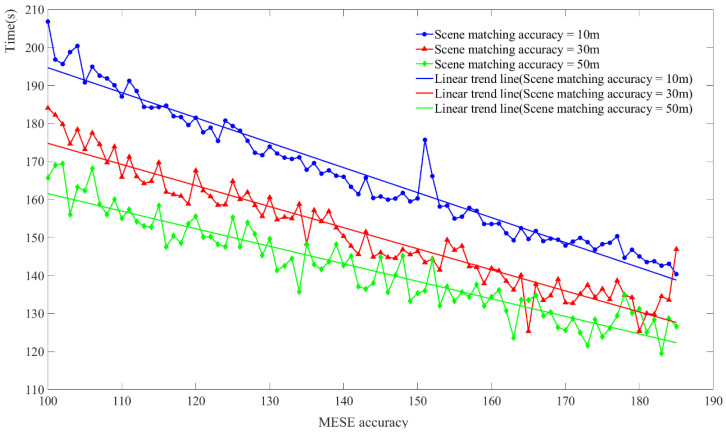
The threshold of the VN+MEMS+SM model and MEMS+SM model.

From Figure 7, the transverse axis demonstrates a variation in the MEMS inertial navigation accuracy, with a range of 100 to 185. The vertical axis is the time when the scene is matched. From the experimental results, it can be seen that the accuracy of the MEMS inertial navigation is inversely proportional to time, and the VN+MEMS+SM model is more suitable for low-precision MEMS equipment and equipment that requires longer scene matching times.

### 3.5. Discussion

This paper examines the relationship between scene matching accuracy, scene matching time, and MEMS inertial navigation accuracy. However, the presence of random errors renders the derivation of an error formula challenging in this model. This paper can only fit the overall error trend through discrete data points, which may not be accurate for more subtle local errors. Nevertheless, the overall trend of the UAV integrated navigation error is applicable, and the accuracy selection of different modules can also be achieved within the range of the average error determination.

**Figure 8:**
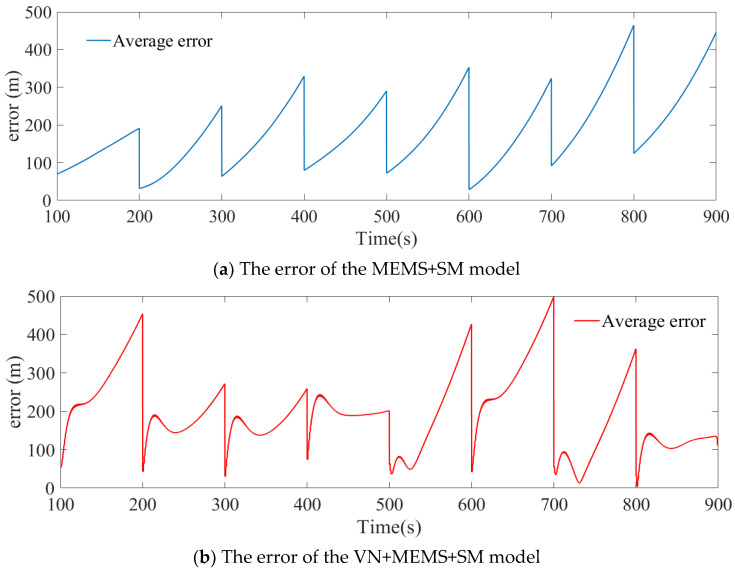
Variation of error with flight time.

In Figure 5, the accuracy of the VN+MEMS+SM model exhibits suboptimal accuracy when the scene matching time is minimal. The reason for this problem is that the divergence of the inertial navigation is exponential, while the divergence of the visual navigation is a one-time functional divergence, and, in the case of a short time, the divergence of the visual navigation is greater than that of the inertial navigation. Figure 8 illustrates the variation trend of the error when the MEMS accuracy is 150, the scene matching accuracy is 50 m, and the scene matching time is 100 s.

Figure 8a shows the variation of the error of the MEMS+SM model with the flight time, and it can be seen that the error changes exponentially in the times without scene matching corrections. Figure 8b shows the variation of the error of the VN+MEMS+SM model with flight time; in the first half, the errors of visual navigation and MEMS inertial navigation together cause the error to rise rapidly, but, because the error of the MEMS inertial navigation changes exponentially, it becomes exponential in the second half. Given that the error associated with the visual navigation is considerably less than that of the MEMS inertial navigation, the exponential trend of the error increase is less pronounced than that observed in the MEMS+SM model. This is why the error of the VN+MEMS+SM model is less than that of the MEMS+SM model when the scene matching time is long.

## 4. Conclusions

To explore the integrated navigation error law between visual navigation, MEMS inertial navigation, and scene matching correction, this article constructs the corresponding visual navigation error model, MEMS inertial navigation error model, and scene matching correction model. This paper constructs the VN+MEMS+SM model and MEMS+SM model through the combination of distinct models. In the experimental part, the average errors of the VN+MEMS+SM model and the MEMS+SM model under different working conditions are determined. Additionally, the correspondence between the scene matching time and the scene matching accuracy under the average error of the MEMS+SM model is established. For example, when the scene matching time is 10 s, the critical values of image matching accuracy the required to achieve the average errors of 10 m, 30 m, and 50 m are about 160 m, 240 m, and 310 m. Finally, the critical value of the scene matching time is calculated when the VN+MEMS+SM model and the MEMS+SM model have equal errors under different MEMS inertial navigation accuracies. The critical value indicates that, when the scene matching time is greater than 135 s, the average error of the VN+MEMS+SM model will be less than that of the MEMS+SM model when the MEMS inertial navigation accuracy is 150 and the scene matching accuracy is 50 m. However, the data used in this model are discrete points and have no further refinement for enhanced accuracy. Due to the existence of random errors, the error outcomes in this model can only fit into curves by discrete points and may not be accurate for more subtle local errors. Nevertheless, this research offers important research significance for the overall trend of integrated navigation and low-cost MEMS integrated navigation.

## Figures and Tables

**Table 1:** Important variables.

Variable Name	Symbol
inertial navigation state from the MEMS	$X_{t}^{Midx}$
the error truth value of the MEMS	$\Delta X_{t}^{Midx}$
triaxial attitude angle	Att
triaxial attitude angle from the MEMS	$Att_{t}^{Midx}$
triaxial velocity vector	V
triaxial velocity value from the MEMS	$V_{t}^{Midx}$
triaxial position coordinates	P
triaxial position coordinates from the MEMS	$P_{t}^{Midx}$
three-axis relative position coordinates calculated by the visual navigation model	$P_{t}^{VN}$
three-axis absolute position coordinate calculated by the scene matching correction model	$P_{t}^{SM}$
triaxial angular velocity	W
triaxial angular velocity from the MEMS	$W_{t}^{Midx}$
three-axis acceleration vector	A
the error value of the MEMS+SM model	$err^{MI+SM}$
the true error of the VN+MEMS+SM model	$err^{MI+VN+SM}$

**Table 2:** Drone model flight angular velocity and accelerometer.

Phase	Angular Velocity—W			Acceleration—A			t/s
	X-axis	Y-axis	Z-axis	X-axis	Y-axis	Z-axis	
1	0	0	0	1	1	0	50 s
2	0	0	0.005π	−0.25π	0	0	200 s
3	0	0	−0.005π	0.25π	0	0	200 s
4	0	0	0	0	0	0	50 s
5	0	0	−0.005π	0.25π	0	0	200 s
6	0	0	0.005π	−0.25π	0	0	200 s
7	0	0	0	0	0	0	100 s

## Data Availability

There are no new data created.
